# Mr. Hill, on the Effect of Cutaneous Eruptions

**Published:** 1805-06-01

**Authors:** Samuel Hill

**Affiliations:** Queen-street, Portsea


### *To the Editors of the Medical and Physical Journal.*

GENTLEMEN,

IT affords me peculiar pleasure to see in your Journal for the present month, that Dr. Jenner's* Communication respecting Herpetic Eruptions impeding or preventing the perfect action of the Vaccine principle, has arrested the attention of some of your Correspondents; and I heartily wish, with those gentlemen, that medical practitioners, would be particular in searching their patients to discover any cuticular disease, with a view to remove it, if practicable, previous to vacciolation. That cuticular complaints, and especially herpetic, when inveterate, do more frequently prevent the proper progress of the vaccine

---

* See Medical and Physical Journal for August 1804.

cine vesicle than s generally known, I am perfectly convinced, having seen in my own practice several instances, where I could not produce the true vesicle on children who had been subject to eruptions on their heads and on different parts of their bodies and extremities; in which cases, the vesicle and areola were completely formed as early as the sixth day. Those children were of course revacciolated, when their eruptions had either been removed by appropriate medical treatment, or had spontaneously disappeared. I was not prepared to ascribe the spurious vesicles produced in those cases, to the presence of herpetic eruptions or psoriasis difusa, until I read Dr. Jenner's interesting paper above alluded to, which has called my attention to the subject, and determined me not to vacciolate in future, when any cuticular complaint exists (except in very slight cases) until it shall have been perfectly cured. I have, in another place, observed, that "Gentlemen could not become all at once perfect Vacciolators;" and might not many, at the commencement of their practice have produced spurious cow-pox, either from the presence of the above disease, or some other cause, and yet thought they had produced the genuine? And, Query, might not many of those children, who, *as we are told,* have had the small-pox subsequent to vacciolation, have had the spurious in lieu of the true cow-pox, when the practice was still in its infancy? I trust more of your Correspondents will turn their thoughts to this subject, it merits the attention of both vaccinist and antivaccinist; the former will see in it an enemy to vacciolation, which, now discovered, will be less dreaded than when concealed. The latter will, I hope, be sufficiently candid to allow, that, it is probable, several of the subjects who, as reported, have had small-pox after Cowpox, *never had the genuine,* but the spurious, which affording no security against small-pox, it ceases to be a matter of wonder that they should take the latter disease.

I rejoice to see the progress of vacciolation on the Continent: in France, as communicated in the Medical Journal for this month by Mr. Ring. I am also happy to state, from the most respectable authority, that only two persons died of the small-pox at Vienna during the year 1804, and they were both imported cases. At Berlin, also the small-pox is done away; and Mr. Shoolbred,* acquaints

* See his Pamphlet.

quaints us that the small-pox is extinct among the Europeans at Calcutta: These important facts have been produced by the vaccine inoculation.

Foreigners have certainly gone more unitedly, steadily, and sensibly forward in the great work of exterminating small-pox, than we have at home; probably they have felt nothing of that envy and prejudice which have started up in England; and to this is owing their extraordinary success. Let us hope that the period is not very distant, when medical gentlemen, in England, will be as unanimous and as successful in the practice of vacciolation, as their medical brethren in Paris, Vienna, Berlin, and in the East Indies.

I will not intrude longer on your valuable Journal than just to state, that in my practice I still continue successful, and have not, to the present day, met with a solitary instance of *even the slightest suspicion* of small pox subsequent to vacciolation,

There being no public institution in this town, for the gratuitous inoculation of cow pox, I have, with a view to benefit the poorer classes of society, and to assist in the great work of exterminating the small pox, lately commenced vacciolating the poor free of expence; and I am extremely happy to add, that many applications have been, and still continue to be made to me, to receive the benefit of that great blessing,

I am, &c.

*Queen-street, Portsea, May* 9, 1805. SAMUEL HILL.

---

